# Cases from the Note-Book of A. Preterre

**Published:** 1868-07

**Authors:** 


### ARTICLE V.

### *Cases from the Note-Book of A. Preterre.*

Translated from l'Art. Dentaire.

### *Hæmorrhage of a Tooth.*

Some time since we were called to Mr. X. an Octogenarian in the neighbourhood of Poissy, to consider the propriety of inserting a set. All the alveolar process of the upper jaw had been completely absorbed, and but a single tooth, one incisor, scarcely adherent to the gum, remained.

We thought the insertion of an artificial denture an easy matter, and recommended the extraction of the sole remaining tooth. This was easily accomplished, without pain, by the employment of no other instrument than our fore finger. A few moments after our patient sat down to dinner, inviting us to join him.

As we were about leaving, Mr. X. noticed that his mouth was full of blood. We immediately sent for perchloride of iron, which we applied on lint over the small cavity left by the removal of the tooth, but without effect. We proposed the actual cautery with red-hot iron, which the patient stoutly refused ; and he even declined to continue the use of the perchloride of iron, which dried his mouth and throat to no purpose.

The ordinary apparatus for compression being inapplicable, on account of the constant trembling of the patient, we resorted to digital compression of the hole left by the extracted tooth. Assisted by the patient's daughter, we kept this up all night. About seven o'clock in the morning we had no further fears of returning hæmorrhage. His health continues excellent and he wears a denture which he says has given him a second youth,

*Disturbance from a Milk-tooth late in life.*

Miss X. thirty-two years of age, came recently to consult us for a tumour at the angle of the lower jaw on the right side. This tumour, which resisted all methods of treatment, had been considered cancerous by several surgeons, and the removal of the jaw had been spoken of as the only treatment to be adopted.

We examined the interior of the mouth with the greatest care, and found that on both sides of the jaw there were two large molars of the first dentition, which checked the evolution of the small molar of which there was only one on either side. On the right side, the first and second large molars were loose, while the molar of the first dentition was fixed. Pressure on the diseased part forced out a considerable quantity of pus.

At first we thought the jaw diseased; but a very minute examination, repeated a second time, proved that the tumour was probably caused by the presence of the roots of a milk tooth lodged in the alveoli. The examination of these roots was very difficult, owing to the state of the parts.

We began by extracting one of the milk teeth, and with a view of dilating the opening and modifying the suppuration, we introduced cotton soaked with a solution of phenic acid, but without result. We then extracted the first large molar of the second dentition, which gave no relief. We now had recourse to small pellets of chloride of zinc which we introduced into the openings left by the extracted teeth.

After the application of these caustics at intervals of ten days, we had the satisfaction of seeing at the extremity of the orifice, a large molar of the first dentition without a crown. We easily removed it. The tumour soon disappeared and the patient was entirely cured.

---

